# Multi-Scale Marine Object Detection in Side-Scan Sonar Images Based on BES-YOLO

**DOI:** 10.3390/s24144428

**Published:** 2024-07-09

**Authors:** Quanhong Ma, Shaohua Jin, Gang Bian, Yang Cui

**Affiliations:** Department of Oceanography and Hydrography, Dalian Naval Academy, Dalian 116018, China; 18642611150@163.com (Q.M.); trighosts@163.com (G.B.); 13998435151@163.com (Y.C.)

**Keywords:** object detection, multiscale, side-scan sonar, deep learning, YOLO

## Abstract

Aiming at the problem of low accuracy of multi-scale seafloor target detection in side-scan sonar images with high noise and complex background texture, a model for multi-scale target detection using the BES-YOLO network is proposed. First, an efficient multi-scale attention (EMA) mechanism is used in the backbone of the YOLOv8 network, and a bi-directional feature pyramid network (Bifpn) is introduced to merge the information of different scales, finally, a Shape_IoU loss function is introduced to continuously optimize the model and improve its accuracy. Before training, the dataset is preprocessed using 2D discrete wavelet decomposition and reconstruction to enhance the robustness of the network. The experimental results show that 92.4% of the mean average accuracy at IoU of 0.5 (mAP@0.5) and 67.7% of the mean average accuracy at IoU of 0.5 to 0.95 (mAP@0.5:0.95) are achieved using the BES-YOLO network, which is an increase of 5.3% and 4.4% compared to the YOLOv8n model. The research results can effectively improve the detection accuracy and efficiency of multi-scale targets in side-scan sonar images, which can be applied to AUVs and other underwater platforms to implement intelligent detection of undersea targets.

## 1. Introduction

Detection and identification of undersea targets have always been the main work of ocean mapping, underwater search and rescue, mine detection, and pipeline surveying. Side-scan sonar can detect and provide high-resolution images of underwater targets in underwater environments with extremely low visibility. It is a common instrument for undersea target detection and plays an important role in searching for wrecked airplanes, shipwrecks, and wrecked people, locating undersea pipelines, and detecting protruding reefs, undersea ores, and submerged mines [1,2,3,4,5,6].

Currently, the identification of undersea targets in side-scan sonar images is still manually based, which is overly dependent on subjective experience and inefficient, which seriously affects its wide application in undersea target detection, especially since it is unable to meet the real-time application requirements of underwater search and rescue and military target detection such as mines [7]. National and international scholars have conducted a lot of research on side-scan sonar image preprocessing, classification and recognition, and target detection algorithms [8,9,10,11,12,13,14,15,16,17,18,19], and have achieved certain results. Due to the complex marine environment, underwater scattering mechanisms, and other factors, there is a large amount of noise in the side-scan sonar image. Therefore, effective suppression of noise while being able to well maintain the edges and details of the image will help to improve the accuracy of target classification recognition and detection. Traditional noise reduction methods mainly focus on the spatial domain, such as mean filters, Wiener filters, etc. [20]. The wavelet transform is one of the typical methods for noise reduction in the frequency domain, which is widely used in side-scan sonar image noise reduction due to its good time-frequency characteristics, multi-resolution analysis characteristics, and sparse representation characteristics [8,21,22,23,24].

The target detection aspect of side-scan sonar images is mainly dominated by deep learning models. Target detection using deep learning models is generally divided into two categories: two-stage detection models and one-stage detection models. Among them, the two-stage detection model first generates a region suggestion network of candidate target boxes; then the category and precise location of the target in the candidate box are further determined. Representative two-stage models include R-CNN [25], Fast-RCNN [26], and Faster-RCNN [27,28]. Although two-stage models achieved high recognition accuracy, their RPN took too long, which reduced the processing speed and made it difficult to meet the real-time requirements for undersea obstacle detection under complex conditions. To reduce the leakage rate and improve the detection efficiency, Joseph Redmon et al. proposed the YOLO network [29,30]. The YOLO network is a typical representative of a one-stage detection model, which uses an end-to-end training method rather than region selection. The one-stage target detection model directly processes the input image while outputting the class and location information of the target object without an explicit candidate region generation step. To improve the training and detection efficiency, Yu-Lin Tang et al. proposed a side-scan sonar shipwreck target detection method based on migration learning to improve the YOLOv3 model [31], but there are still problems, such as the high rate of missed alarms for small targets, and the detection speed cannot meet the real-time requirements. Wang et al. [32] introduced an attention mechanism converter and a convolutional block attention module based on the YOLOv5 model to improve the detection of small-target objects in the side-scan sonar images. Although the above models improve the detection accuracy and overall detection efficiency of small-scale targets, in the actual marine environment, the size and distribution of objects are usually very complex. How to simultaneously take into account the detection accuracy and detection efficiency of multi-scale targets to improve the detection performance in complex marine situations is the focus of this paper to solve the problem.

Based on the above analysis, an improved model based on YOLOv8 is proposed in this paper. The model applies to the task of target detection in side-scan sonar images, and during the training process, data preprocessing methods are used to optimize the dataset so as to enhance the robustness of the network to be able to accurately and quickly identify targets of different sizes in the complex marine environment. The main research and innovations of this paper can be summarized as follows:

(1) Aiming at the characteristics of underwater images with severe noise, the data set is preprocessed using the wavelet transform method to improve the generalization and robustness of the model.
(2) The feature fusion structure of the original model is replaced with Bifpn, which can capture feature information at different scales more efficiently and thus improve detection accuracy.
(3) To enhance the model’s ability to focus on target features, the EMA module is used, which is effective in obtaining clearer multi-scale features.
(4) Finally, we introduce the Shape_IoU loss function, which takes into account the characteristics of the bounding box, such as shape and scale, to make the bounding box regression more accurate, thus improving the accuracy of target detection.

The rest of this study is organized as follows: in Section 2, we focus on the operational flow of underwater target detection based on AUV-carrying side-scan sonar and analyze specific improvement strategies for the model. Section 3 focuses on the general analysis of the dataset and training strategy, as well as the design of the experiments and discussion of the results. Section 4 provides the final conclusions and directions for future work.

## 2. Materials and Methods

**Figure 1:**
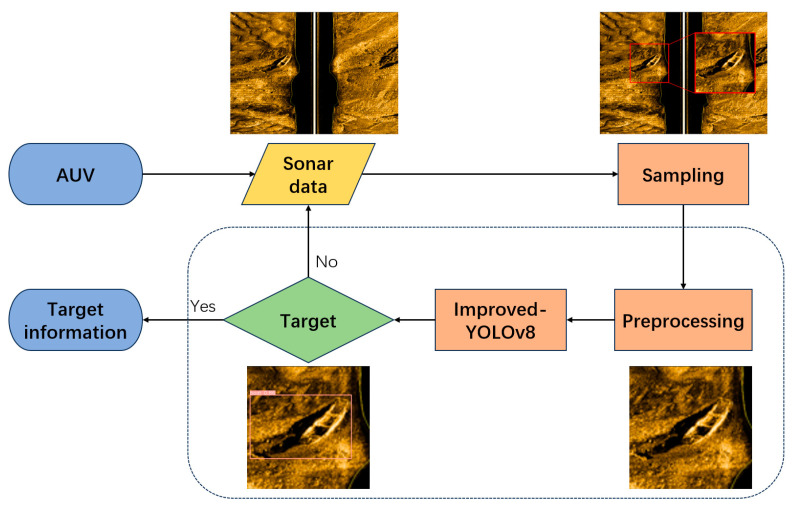
Operational flowchart of AUV underwater target detection.

The operation flowchart of underwater target detection using AUV-carried side-scan sonar is shown in Figure 1, which mainly includes sampling of side-scan sonar data, preprocessing, and obtaining underwater target information using the BES-YOLO. This paper mainly focuses on image preprocessing and BES-YOLO.

### 2.1. Preprocessing

**Figure 2:**
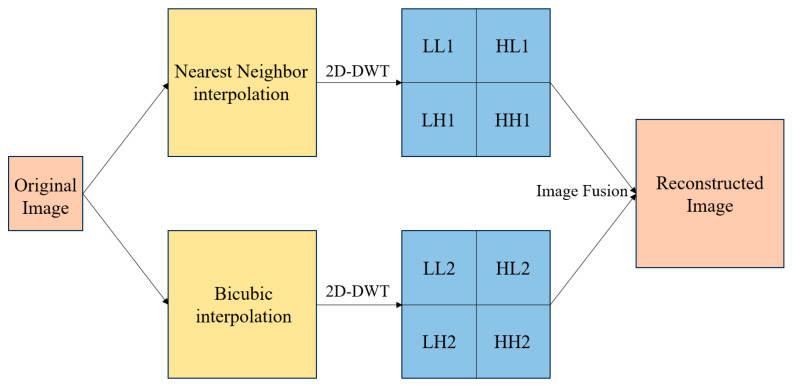
Flowchart of wavelet transform preprocessing.

A wavelet transform is a localized transform method for image processing based on time, space, and frequency, which can decompose a signal or image into components of different frequencies and different times. This decomposition method is very useful for extracting local features of a signal or image. In image processing, the wavelet transform can decompose an image into multiple sub-images of different scales and orientations to extract the detailed information of the image. After the low-frequency and high-frequency parts are processed differently, they are then reconstructed to get richer detail information and achieve the purpose of image enhancement. The specific operation flow is shown in Figure 2.

**Figure 3:**
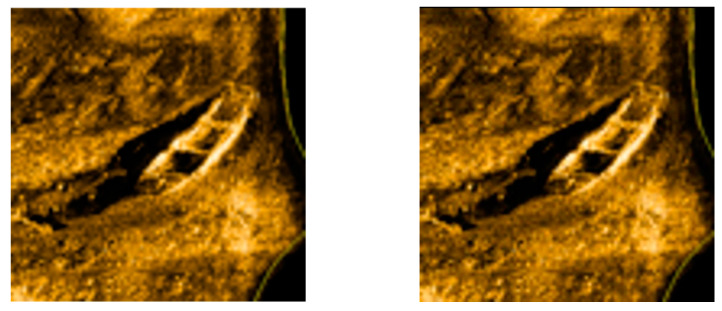
Wavelet transform processing effect.

In the pre-processing process of the side-scan sonar image, we perform the nearest neighbor interpolation on the low-resolution image, and the image obtained from the interpolation is transformed by discrete wavelet using a Haar wavelet, which enables the original image to be completely transformed into its low-frequency coefficients without any loss of information, i.e., the low-frequency portion contains all the information of the original low-resolution image. Next, the original image is interpolated bicubically and then decomposed by a Haar wavelet to extract the high-frequency coefficients in three directions. After completing the integration of the low-frequency components with the three superimposed high-frequency components, the high-resolution image is reconstructed using wavelet inversion. This reconstruction process not only fully utilizes all the information in the low-resolution image but also combines the relevant high-frequency information to ensure that the high-frequency detail part is better represented in the reconstruction process. In this way, the quality of the reconstructed image is improved, especially in the high-frequency details, so that the whole image presents higher clarity and richer details. As shown in Figure 3, the left side is the original image, and the right side is the processed image.

In the reconstruction process, the low-frequency part of the wavelet inverse transform is no longer used directly in the original image but according to the characteristics of the Haar wavelet, so that all the energy of the low-resolution image is gathered in the low-frequency part, which not only corrects the mismatch between the high and low-frequency coefficients of the wavelet inverse transform but also avoids the gray scale shift, so that the reconstructed image is enhanced.

### 2.2. BES-YOLO

The YOLOv8 model structure consists of four parts: input, Backbone, Neck, and output, which contain the Conv module, C2f module, SPPF (spatial pyramid pooling-fast) module, etc. YOLOv8 innovatively introduces the C2f structure, which is an important part of the learning of residual features, and is able to efficiently capture rich gradient flow information. At the last layer of the model’s backbone network, the SPPF structure is introduced, which captures information with sensory field sizes of 5, 9, and 13, respectively, through a series of consecutive 5 × 5 convolutional kernel max-pooling operations. Subsequently, these SPPF-processed feature layers are fused with the unprocessed feature layers to synthesize feature information at different scales to improve the performance of the model. Backbone mainly consists of 5 Conv, 4 C2f, and 1 SPPF structures to extract the generic features of the target; Neck is located between Backbone and Head and contains 4 C2f, 2 Conv, and two Upsample, which aims to further enrich the diversity of features to improve the robustness of the model; Head is the output end, which serves to complete the output of the target detection results.

**Figure 4:**
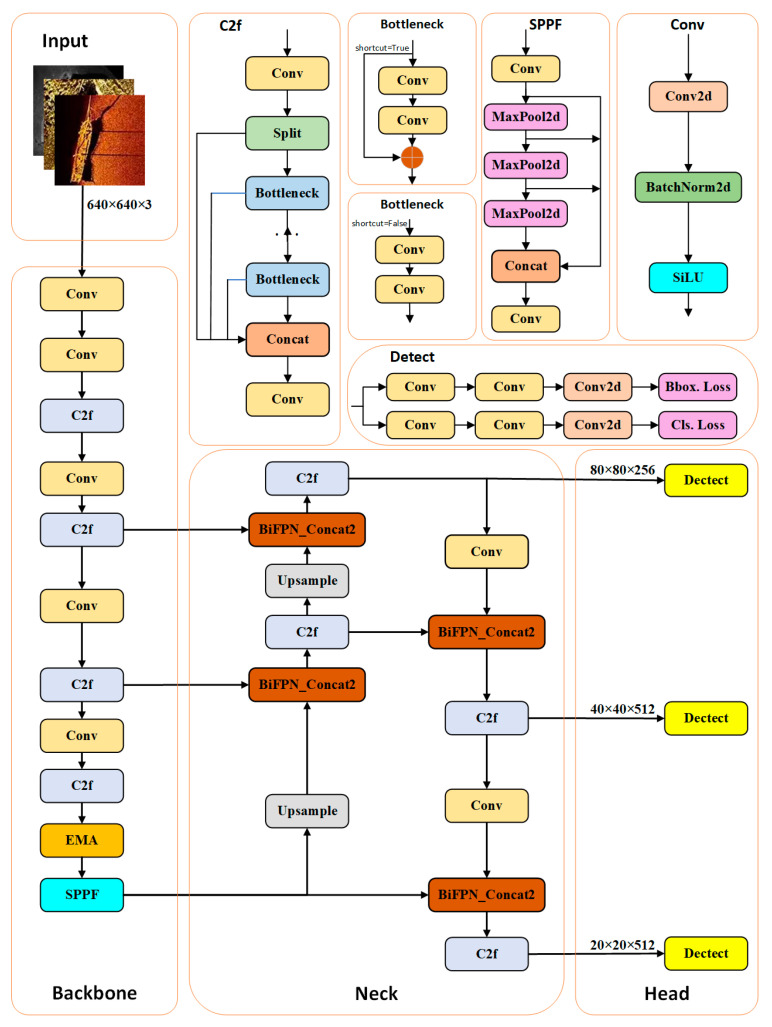
BES-YOLO network structure diagram.

In this paper, we propose a BES-YOLO model that utilizes a bi-directional feature pyramid network (Bifpn) in combination with the efficient multi-scale attention module (EMA) and applies the Shape_IoU loss function to meet the challenge of multiscale target detection. The network structure is shown in Figure 4. We first introduce EMA in the backbone. The EMA mechanism can fuse contextual information from different scales, capture cross-dimensional interactions, and establish dependencies between dimensions so that the huge local receptive fields of neurons can efficiently obtain clearer multiscale features, which helps the network reduce the influence of interfering factors in the image. The introduction of Bifpn allows information to propagate bidirectionally between different resolution levels for a better fusion of multi-scale information. This improves the detection performance of multi-scale objects and the contextual understanding of objects, which helps reduce false or missed alarms. Finally, we introduce the Shape_IoU loss function to be able to more accurately evaluate the performance of the target detection model and optimize the target detection model. The Shape_IoU loss function can guide the model to pay more attention to the shape and proportion of the bounding box during the adjustment process, thus improving the overall performance of the model.

#### 2.2.1. Bifpn

**Figure 5:**
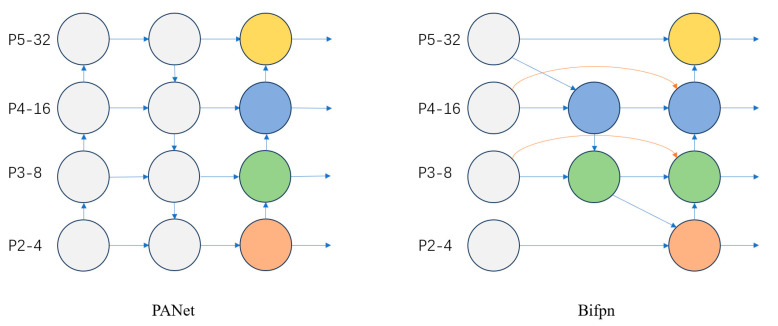
Structure of PANet with Bifpn.

To better fuse multiscale features in side-scan sonar images, the PANet in YOLOv8 is replaced with Bifpn. Compared to other structures, Bifpn can efficiently fuse features without increasing the computational cost. The main idea behind Bifpn [33] is to construct a feature pyramid by utilizing the information from both bottom-up and top-down directions while using a repetitive weighting fusion method at each pyramid level. By utilizing information from both directions, Bifpn can fuse different levels of features and enhance the accuracy and generalization of the model, thus improving the target detection performance. Compared to the PANet feature fusion network, Bifpn simplifies the network structure by removing unidirectional input nodes. Connecting the original input nodes and output nodes of the same layer allows the feature graph of the layer to be better preserved and utilized in the feature fusion process. This enhances the information transfer and fusion ability of the same-layer feature graphs and improves target perception and recognition, and by repeating the process, the network gradually fuses more layers of features, resulting in a more comprehensive and semantically expressive feature representation rather than a simple stacking or addition of feature graphs. Due to its complex connectivity patterns, an accurate training strategy needs to be designed. By combining Bifpn into BiFPN_Concat2 modules, we set learnable parameters and learning weights for different branches and apply the Concat operation to the two-branch and three-branch combinations of feature maps, respectively. The structure of Bifpn is shown in Figure 5.

YOLOv8’s replacement of Bifpn allows for better multi-scale information fusion. The introduction of Bifpn allows information to propagate bi-directionally between different resolution levels, resulting in a better fusion of multi-scale information. This helps the model understand targets of different sizes more comprehensively and improves the detection performance of multi-scale objects. It also improves the contextual understanding of the objects and helps to reduce false or missed alarms.

#### 2.2.2. EMA

The attention mechanism has been widely used in computer vision, and its basic principle is to suppress useless feature information while reinforcing useful feature information to enable the model to focus more adaptively on important regions in the image. The traditional SE (squeeze-and-excitation) [34] attention mechanism mainly focuses on constructing interdependencies between channels, with less consideration for spatial features. CBAM (convolutional block attention module) [35] improves the model performance by effectively combining spatial attention and channel attention model performance, but it can only effectively capture local information and is difficult to establish long-distance channel dependencies.

The efficient multiscale attention (EMA) mechanism [36], as an innovative parallel attention mechanism, effectively improves the processing speed and performance of the model through its unique parallel structure. Compared with the traditional convolutional neural network (CNN), EMA shows higher efficiency in processing large-scale data, and its parallel convolutional feature makes the model training more rapid.

**Figure 6:**
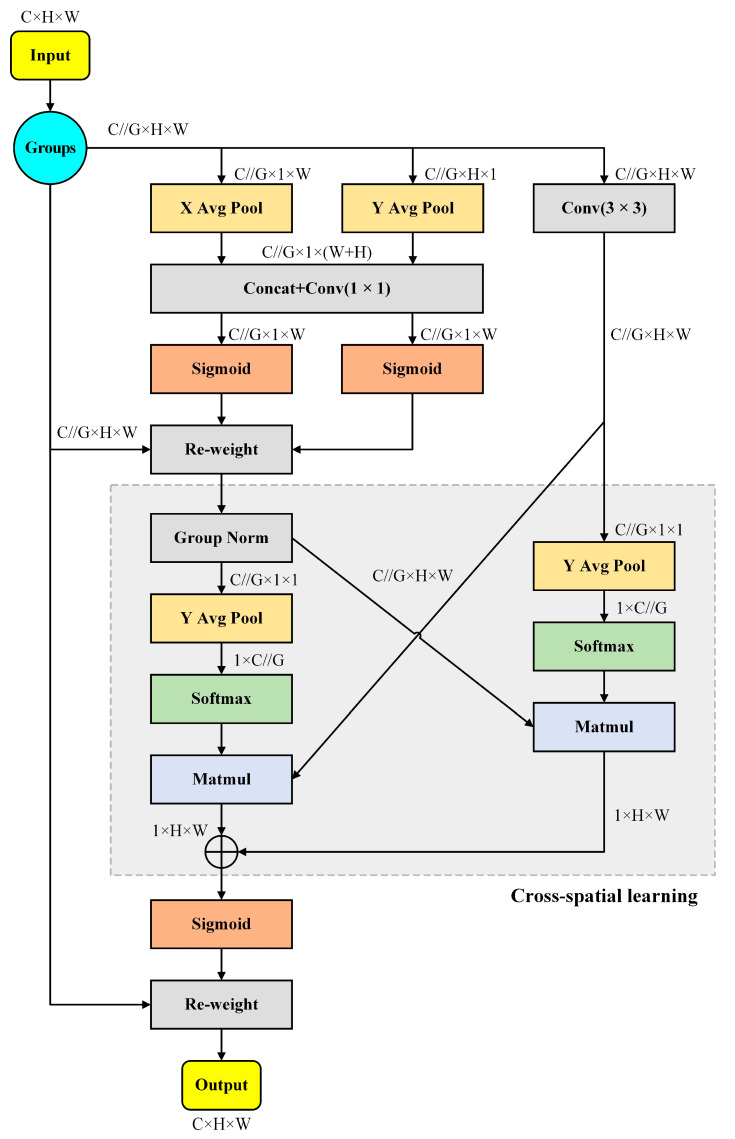
Structure of the EMA attention mechanism.

The core of the EMA mechanism lies in its ability to process features at different scales simultaneously, which enhances the model’s ability to perceive information at different scales and improves the accuracy of the model. During feature processing, EMA not only encodes the inter-channel information for adjusting the importance of each channel, but also finely preserves the details of the spatial structure within the channel, thus ensuring the completeness and accuracy of the information. In addition, the EMA mechanism introduces a cross-space information aggregation strategy to optimize the interaction between features. This enables the model to aggregate information from different spatial locations more effectively, further enhancing the model’s ability to understand and process complex feature relationships. Compared with CBAM and SE, EMA not only has higher performance but is also more efficient in terms of the required parameters. Therefore, in this paper, the EMA mechanism is added to the backbone network of the YOLOv8 baseline model. The EMA structure is shown in Figure 6.

#### 2.2.3. Shape_IoU

**Figure 7:**
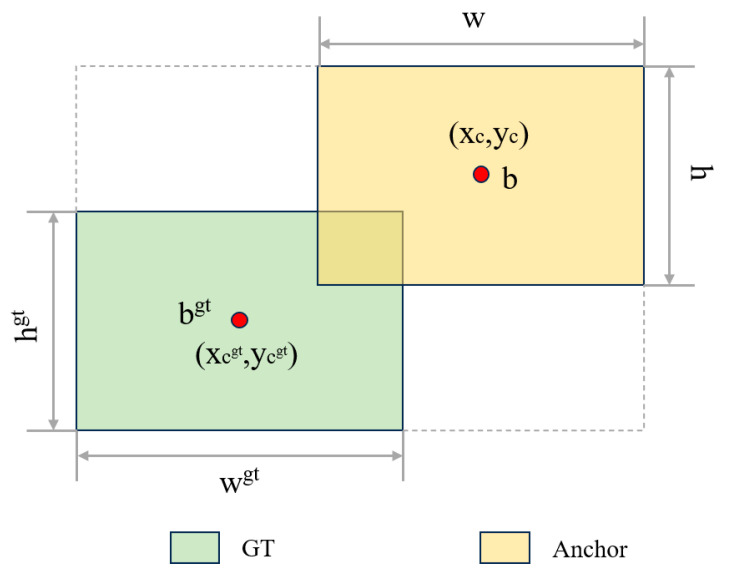
Schematic diagram of Shape-IoU principle.

Due to the significant differences in target scales and the existence of many small targets in side-scan sonar images, current edge regression methods usually focus on considering the geometric relationship between the real frame (GT frame) and the predicted frame and calculate the loss by taking into account factors such as the relative positions and relative shapes of the frames. However, these methods ignore the influence of the frame’s inherent properties, such as its own shape and scale, on the frame regression, resulting in low detection accuracy when processing side-scan sonar images. To solve this problem, a border regression method focusing on the shape and scale of the border itself [37], i.e., the Shape-IoU (shape-intersection-using ratio) loss function, is introduced. The method first analyzes the characteristics of border regression and concludes that the shape factor and scale factor of the border itself will have an impact on the regression results. Shape-IoU can calculate the loss by focusing on the border’s own shape and its own scale, so as to make the regression of the border more accurate, and the Shape-IoU is shown in Figure 7.

(1) $$IoU=\frac{\left|B\cap B^{gt}\right|}{\left|B\cup B^{gt}\right|}$$

(2) $$ww=\frac{2\times {\left(w^{gt}\right)}^{scale}}{{\left(w^{gt}\right)}^{scale}+{\left(h^{gt}\right)}^{scale}}$$

(3) $$hh=\frac{2\times {\left(h^{gt}\right)}^{scale}}{{\left(w^{gt}\right)}^{scale}+{\left(h^{gt}\right)}^{scale}}$$

(4) $$distance^{shape}=hh\times {\left(x_{c}-x_{c}^{gt}\right)}^{2}/c^{2}+ww\times {\left(y_{c}-y_{c}^{gt}\right)}^{2}/c^{2}$$

(5) $$\Omega^{shape}=\sum_{t=w,h}{\left(1-e^{-\omega t}\right)}^{\theta },\;\theta =4$$

(6) $$\left\{\begin{array}{l} \omega_{w}=hh\times \frac{\left|w-w^{gt}\right|}{\max\left(w,w^{gt}\right)}\\ \omega_{h}=ww\times \frac{\left|h-h^{gt}\right|}{\max\left(h,h^{gt}\right)} \end{array}\right.$$

where scale is the scale factor, which is related to the scale of the detected target in the dataset, *w^gt^* and *h^gt^* are the length and width of the GT frame, *x_C_^gt^* and *y_C_^gt^* are the coordinates of the center point of the GT frame, *w* and *h* are the length and width of the a priori frame (Anchor), *x_C_* and *y_C_* are the coordinates of the center point of the a priori frame, and ww and hh are the weight coefficients of the horizontal and vertical directions, respectively, whose values are related to the shape. Its corresponding regression loss of the frame is as follows:

(7) $$L_{Shape-IoU}=1-IoU+distance^{shape}+0.5\times \Omega^{shape}$$

where the *IoU* loss function, *distance^shape^* distance loss function, and Ω*^shape^* shape loss function. As shown in the equation.

## 3. Results and Discussion

### 3.1. Experimental Environment and Datasets

The study was conducted on the Windows 11 operating system, utilizing the Pytorch deep learning framework. The hardware used for the experiments included an Intel Core i7-14650HX CPU, NVIDIA GeForce RTX 4060 Laptop GPU, and 64 GB of RAM. Additional details on the hardware and software configurations used in the experiments are provided in Table 1. To improve training efficiency while ensuring model training effectiveness, the ratio of the training set to the validation set in the dataset was set to 8:2; no pre-training weights were used in the training of the model. The optimizers commonly used in the process of training the model are SGD, Adam, RMSProp, etc. To take into account the convergence speed and stability of training. The SGD optimizer was used in the experiments. The SGD optimizer is used in the training process, and before the start of training, the number of rounds of warm-up training is 3, the learning rate is set to 0.01, and the learning rate momentum is set to 0.937; the number of rounds of training is set to 200 rounds, and according to the configuration of the computer, the batch size is set to 16, the training rate is set to 0.01, and the training rate is set to 0.937. The relevant parameters in the training process are shown in Table 2.

**Figure 8:**
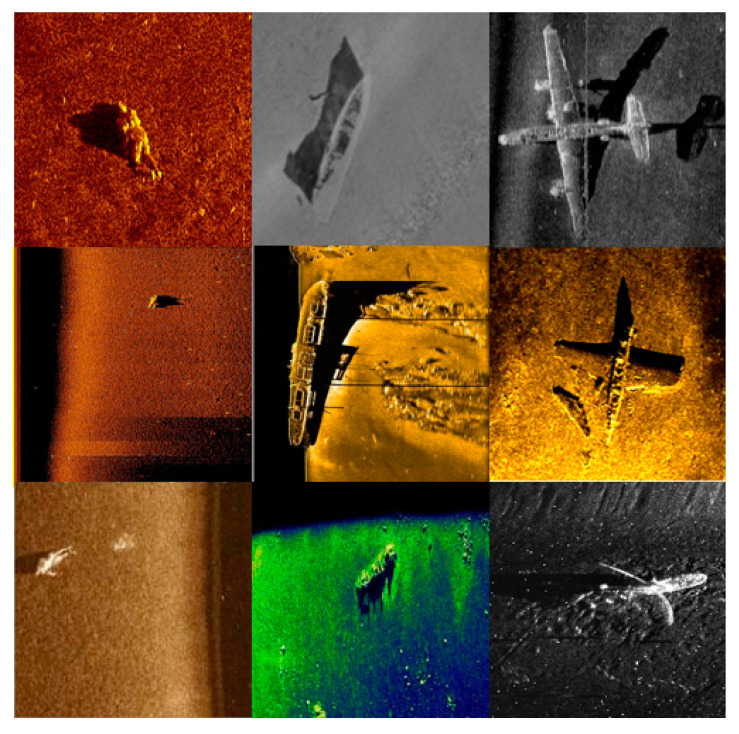
Selected data sets.

The dataset used for experiments in this paper mainly consists of side-scan sonar shipwreck images, airplane wreckage images, and human image data. The side-scan sonar dataset consists of three types of side-scan sonar images, totaling 1584 images, obtained by various hydrographic departments and domestic manufacturers using mainstream domestic and foreign side-scan sonar instruments and equipment such as Klein3000, EdgeTech4200, Yellowfin, and SS900 series, which are measured in the regions of the Yellow Bohai Sea, the East Sea, and the South China Sea in China, as well as collected on the network. Some samples are shown in Figure 8.

**Figure 9:**
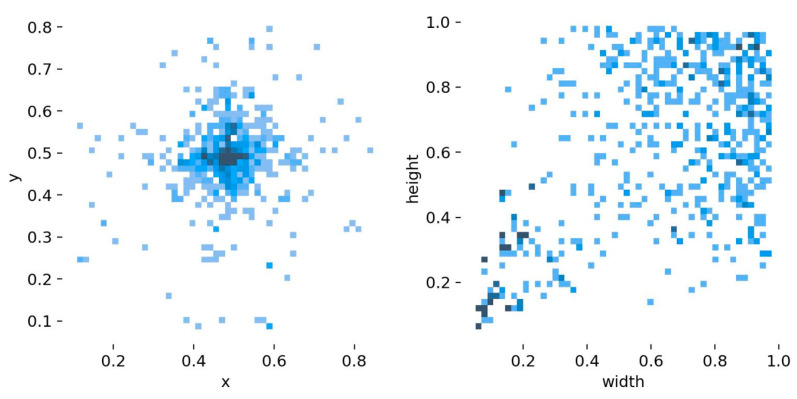
Target distribution and sizing.

To better analyze the characteristics of the targets in the dataset, the distribution of the location of the targets in the picture as well as the aspect ratio of the targets, such as shipwrecks and airplanes, relative to the picture were counted, in which the depth of the color represents the number of targets, and the specific statistics are shown in Figure 9.

### 3.2. Evaluation Metrics

In this paper, the performance of the optimization model is evaluated by the following coefficients: precision *P* (precision), recall *R* (recall), average precision *AP* (average precision), and mean average precision mAP (mean AP).

Precision and Recall: In the classification task of predicting whether an image contains a package or not, the four elements in Precision and Recall can be interpreted as follows: *TP* (true positive): positive samples are correctly labeled as positive samples in the prediction results of positive samples; *TN* (true negative): the negative sample is correctly labeled as a negative sample in the prediction result of the negative sample; *FP* (false positive): the positive sample is incorrectly labeled as a negative sample in the prediction result of the positive sample; *FN* (false negative): the negative sample is incorrectly labeled as a positive sample in the prediction result of the negative sample, calculated as.

(8) $$P=\frac{TP}{TP+FP}$$

(9) $$R=\frac{TP}{TP+FN}$$

Average precision: the geometric meaning of average precision *AP* is the area corresponding to the PR curve as in Equation (9), where the integral can be approximated using interpolation and summation.

(10) $$AP=\int_{0}^{1}p\left(r\right)dx$$

Average precision mean: the *mAP* type used in this paper is: mAP@0.5 and mAP@0.5:0.95. mAP@0.5 (average precision coefficient of the total category) characterizes the precision and the average of all the images used to calculate each category when the IoU is set to 0.5; and mAP@0.5:0.95 denotes the average precision coefficient of the total category when the IoU is in the range of 0.5 to 0.95, which is calculated as follows:

(11) $$mAP=\frac{\sum_{i-1}^{N}AP_{i}}{N}$$

### 3.3. Performance Evaluation

**Figure 10:**
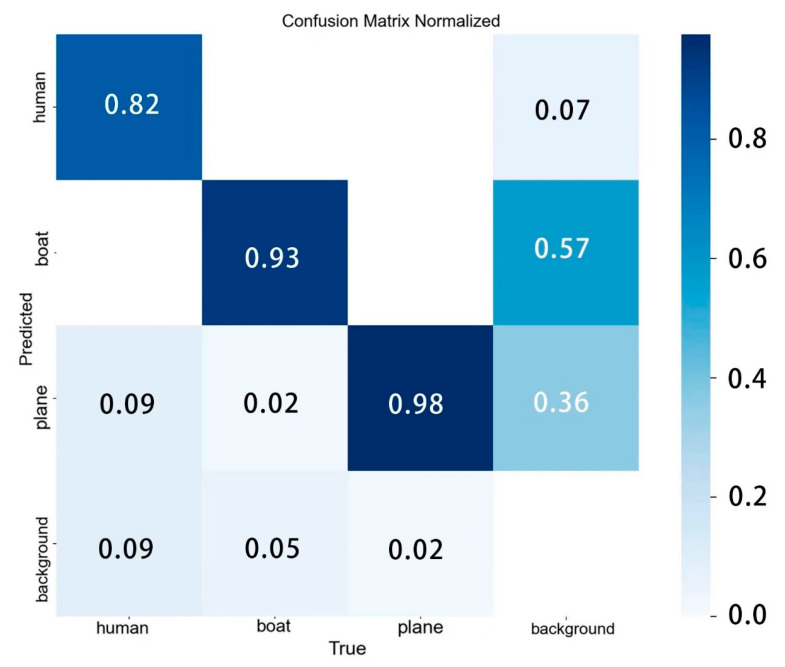
Confusion matrix plot for BES-YOLO model.

**Figure 11:**
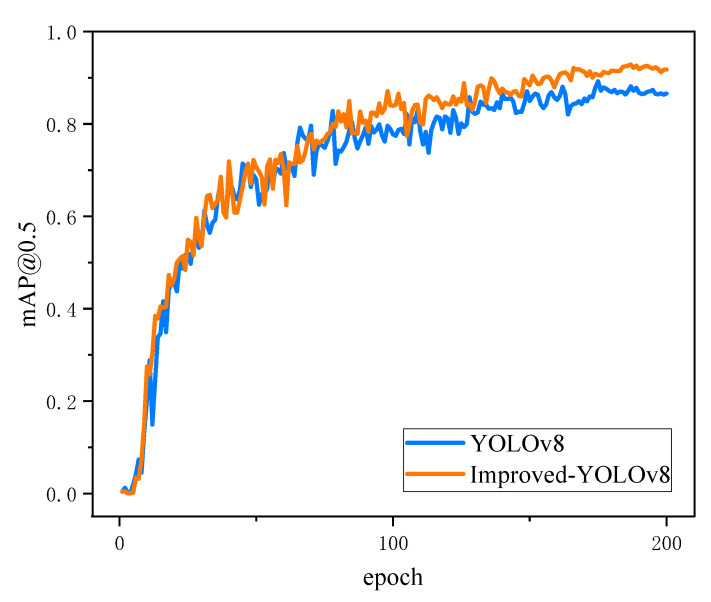
Comparison of mAP curves of two models.

Figure 10 shows the confusion matrix for the improved model. The horizontal axis represents the true values, and the vertical axis represents the predicted values, and it can be seen that most of the predicted values correspond to the true values. Figure 11 shows a comparison of the mAP@0.5 curves of the improved model and the baseline model, and it can be seen that the improved curves are higher than the baseline model. Based on the data of these two figures, it is found that the improved model has better prediction performance.

**Figure 12:**
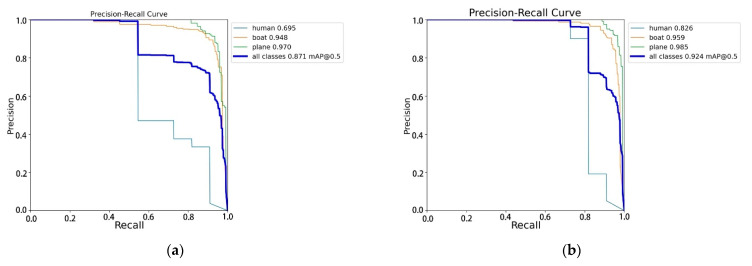
(**a**,**b**) Comparison of the PR graphs of the two models.

A PR curve is a curve with precision on the vertical axis and recall on the horizontal axis. Usually, precision and recall are mutually constrained metrics. Therefore, plotting PR curves allows one to explore the comprehensive performance of the model. The observation curve can reflect the performance of the deep learning model. The PR curve plots in Figure 12 illustrate the experimental results for YOLOv8 and BES-YOLO under the same conditions. The figures show the mAP@0.5 in each category as well as the overall mAP@0.5. As can be seen from the graphs, the improved model increases the mAP from 87.1% to 92.4%, an improvement of 5.3%. It is worth noting that in the YOLOv8 assay, the mAP for the “human” category, which has a small target size, was only 69.5%, whereas it reached 82.6% in BES-YOLO. This is a significant enhancement compared to the baseline model for this particular category. The mAP of the other two categories also improved.

**Figure 13:**
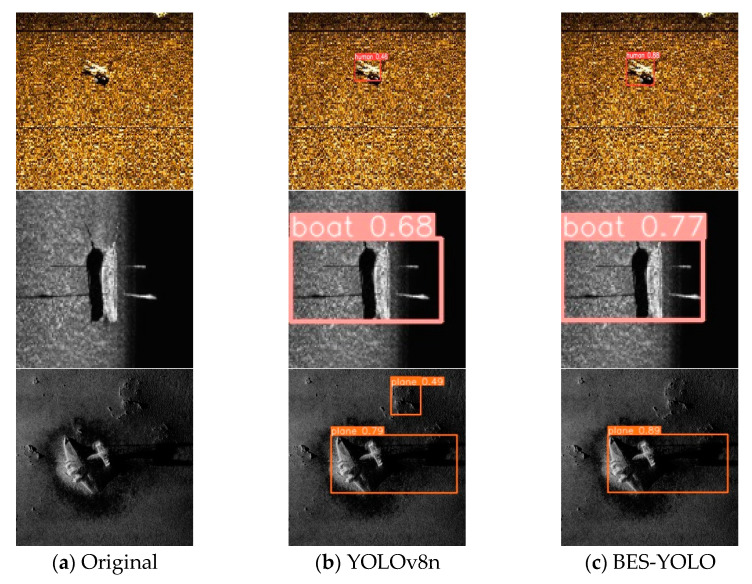
Comparison of results.

Figure 13 shows the prediction results. The location of the prediction box for target detection by the improved model is more accurately focused on the target itself, and the confidence scores are all improved to some extent, while we can notice that in the third set of images regarding the prediction of the airplane wreckage, the original model misjudges the undersea reef in the background as an airplane, whereas this problem does not occur in the improved model. The comparison clearly shows that the improved model achieves more accurate object localization and some degree of confidence score improvement.

To verify the effectiveness of the proposed model, this paper compares the model with mainstream target detection models, including SSD, Faster-RCNN, YOLOv5s, YOLOv7, and YOLOv8n. The experimental results are shown in the table for comparison. Table 3 shows the AP values of the different models for various target types as well as the mAP@0.5 values of the final model. it can be seen that although the detection performance of Faster-RCNN may be slightly better, as a two-stage detection model, the number of parameters increases more relative to the one-stage detection model, and the computation amount increases significantly.YOLOv5s and YOLOv7 have relatively poorer detection results in the case of small target sizes and dense distributions.YOLOv8 has relatively poor detection results in the case of small targets of the class “human” type of small targets. The improved YOLOv8 realizes an overall improvement in detection performance. It not only shows significant improvement in detecting some challenging small targets but also improves the detection of large targets. Based on the above findings, the proposed model in this paper outperforms other models in accomplishing the detection task in the undersea multi-scale target detection task.

### 3.4. Ablation Study

This section describes the whole process of the improvement experiment and evaluates the impact of each improvement module on the overall performance.

According to the data in Table 4, the Neck part of YOLOv8n is reconstructed using the Bifpn concept, and feature fusion is performed through Bifpn, which allows the information to be propagated bi-directionally between different resolution levels and better fuses multi-scale target information. The mAP@0.5 of the model is improved by 3.9%, and mAP@0.5:0.95 is improved by 0.9%. At the same time, the EMA module is added to the backbone of the baseline model to fuse contextual information at different scales, and effective channel descriptions are learned without channel dimensionality reduction during the convolution operation, enabling the model to produce better pixel-level attention to high-level feature maps, resulting in an increase of 3.5% in mAP@0.5 and 1.1% in mAP@0.5:0.95, respectively. Finally, we replace the loss function of the original baseline model with Shape-IoU to further improve the detection accuracy of the model, which is shown by the data to increase the mAP@0.5 by 2.7% and mAP@0.5:0.95 by 2.5%. Overall, the BES-YOLO model combines the Bifpn, the EMA attention mechanism, and the Shape-IoU loss function, which far exceeds the original YOLOv8 model in detection accuracy. On the same dataset, the BES-YOLO model achieves a 5.3% increase in mAP@0.5 and a 4.4% increase in mAP@0.5:0.95, indicating that the corrected model has significantly improved the detection accuracy of multi-scale targets. For a comparison of the accuracy between the original model and the improved model, please refer to Figure 11.

The following ablation experiments are performed on the attention mechanism and the loss function, respectively.

#### 3.4.1. Attention Mechanisms

The attention mechanism plays a crucial role in the target detection model, which significantly improves the detection performance of the model. The addition of the attention mechanism not only improves the model’s ability to extract effective features but also improves the accuracy of the detection, reduces the risk of false and missed detections, and also optimizes the computational resource allocation of the model to improve the processing speed. Therefore, we chose two attention mechanisms to compare with the EMA in this paper. The experimental results show that EMA improves the detection of both small as well as significant targets and other multi-scale targets, with an increase of about 3.5% in mAP@0.5 compared to the original network (Table 5).

#### 3.4.2. Loss Function

Shape_IoU is used as a loss function in the BES-YOLO model, and to verify that this loss function has a better enhancement effect on the model’s accuracy in detecting the target, the same model after using different loss functions will be compared. The YOLOv8n-based model is used for comparison experiments with 200 rounds of training. The experimental results are shown in Table 6.

As can be seen from Table 6, compared with other loss functions, Shape_IoU has the greatest improvement in the detection effect of the model under different scale targets, with mAP@0.5 of 89.8%, which is 2.7%, 2.5%, and 1.1% higher than the other three models, respectively, and at the same time, there is an increase in the class of small-scale targets by 7.2% compared with the AP of the original YOLOv8 that uses the CIoU loss function, which indicates that the loss function Shape_IoU improves the detection accuracy of small targets in complex marine environments by a large amount without causing a loss of detection accuracy of medium and large targets.

## 4. Conclusions

The proposed multi-scale underwater target detection model BES-YOLO integrates an EMA attention mechanism, incorporates Bifpn, and employs the Shape_IoU loss function, leading to a substantial enhancement in the model’s detection accuracy. Particularly focused on overcoming the challenge of achieving high accuracy for multi-scale targets concurrently, the algorithm exhibits remarkable improvements in target detection performance across various scales, especially excelling in detecting small targets. By implementing two-dimensional discrete wavelet decomposition and reconstruction for data augmentation, the model’s robustness is significantly boosted, enabling it to deliver solid performance even with limited resources. Experimental findings reveal that the enhanced YOLOv8 model achieves a 92.4% mAP@0.5 in side-scan sonar image target detection, showcasing noteworthy enhancements in mAP@0.5:0.95 metrics by 5.3% and 4.4%, respectively. This notable advancement underscores the algorithm’s efficacy in multi-scale underwater target detection, offering technical support for precise underwater target detection tasks in practical scenarios like AUV platforms and is very beneficial to improving the accuracy of real-time underwater target detection. Nonetheless, there exist several areas for further enhancement. Firstly, improving the dataset quality is imperative, considering the current dataset suffers from small and uneven sample sizes. Future efforts should focus on acquiring more videos and images for comprehensive model training and testing. Addressing the imbalance in the number of individual detection categories in the dataset by rebalancing sample sizes is also crucial. Secondly, enhancing side-scan sonar images affected by low contrast, blur, speckle noise, and grayscale distortion using image enhancement algorithms can refine the model’s generalization capabilities. Lastly, optimizing the model’s processing to reduce size and computational complexity is recommended. Employing model pruning techniques to eliminate redundant network connections can enhance speed and efficiency to tailor towards lightweight models suitable for embedded devices on experimental platforms and future applications.

## Figures and Tables

**Table 1:** Experimental configuration.

Name	Parameters
Operating system	Windows11
CPU	Intel Core i7-14650HX
GPU	NVIDIA GeForce RTX 4060 Laptop
CUDA	11.7
Pytorch	1.13.1
Python	3.8

**Table 2:** Training parameters.

Parameter Name	Parameter Information
lr0	0.01
momentum	0.937
warm_up	3
batch_size	16
imgsz	640

**Table 3:** Comparison of different model.

Detection Network	Human	Boat	Plane	mAP@0.5
	AP	AP	AP	
Faster-RCNN	68.4	93.3	95.5	85.7
SSD	66.2	87.6	89.7	81.2
YOLOv5s	57.7	77.3	79.1	71.4
YOLOv7	58.1	75.2	77	70.1
YOLOv8n	69.5	94.8	97	87.1
BES-YOLO	**82.6**	**95.9**	**98.5**	**92.4**

**Table 4:** Comprehensive ablation experiments.

Bifpn	EMA	Shape_IoU	mAP@0.5	mAP@0.5-0.95
			87.1	63.3
√			91	64.2
	√		90.6	64.4
		√	89.8	65.8
√	√		91.5	64.5
√		√	91.3	65.8
	√	√	91.6	66.1
√	√	√	**92.4**	**67.7**

**Table 5:** Demonstrates the comparison of different attention mechanisms.

Detection Network	Human	Boat	Plane	mAP@0.5
	AP	AP	AP	
YOLOv8	69.5	**94.8**	97	87.1
YOLOv8+SE	72.4	93.4	97.4	87.7
YOLOv8+CBAM	77.8	92.5	96.8	89.1
YOLOv8+EMA	**79.6**	94	**98.1**	**90.6**

**Table 6:** Comparison of loss functions.

Detection Network	Human	Boat	Plane	mAP@0.5
	AP	AP	AP	
CIoU	69.5	94.8	97	87.1
GIoU	69.6	93.9	**98.4**	87.3
DIoU	72.7	**95.4**	95	88.7
Shape_IoU	**76.7**	94.6	98	**89.8**

## Data Availability

Access to the data will be considered upon request by the authors.
